# Finger gangrene as initial manifestation of esophageal squamous cell carcinoma: a case report

**DOI:** 10.3389/fonc.2025.1681048

**Published:** 2025-11-19

**Authors:** Kai Chen, Qiongzhi Jiang, Juju Zhou, Lingya Yang, Daquan Zhong, Tiantian Zhai

**Affiliations:** 1 Department of Radiation Oncology, Cancer Hospital of Shantou University Medical College, Shantou, Guangdong, China; 2 Cancer Center, The First People's Hospital of Foshan, Foshan, Guangdong, China

**Keywords:** esophageal squamous cell carcinoma, finger gangrene, phalangectomy, immunochemotherapy, radiochemotherapy

## Abstract

Esophageal cancer exhibits a high incidence in the Chaoshan region of south China, an area characterized by distinctive dietary habits prominently featuring the consumption of preserved pickles and hot tea. Common metastatic sites of esophageal cancer include the lungs, liver, brains, and bones, while occurrence in the distal ends of the limbs is exceptionally rare. Here we present a case of a 62-year-old man from China’s Chaoshan region presented with progressive right ring finger pain, swelling, and gangrene. After the amputation of digit, histopathology unexpectedly identified well-differentiated esophageal squamous cell carcinoma (ESCC) with positive margins. A comprehensive retrospective evaluation of medical history identified previously undocumented intermittent dysphagia and mild thoracodorsal pain, postdating the initial presentation of digital symptom by one months. The Esophageal Cancer Multidisciplinary Team confirmed the diagnosis of mid-thoracic ESCC at stage T4bN2M1 according to the AJCC(The American Joint Committee on Cancer, AJCC) 8th edition criteria based on further examinations. Following a multidisciplinary approach encompassing digital amputation, immunochemotherapy, and localized radiotherapy to the primary lesion, the disease achieved partial response according to RECIST(Response Evaluation Criteria in Solid Tumors, RECIST) 1.1 criteria.This case highlights the diagnostic challenges of rare metastatic presentations in ESCC, and the critical importance of recognizing atypical symptoms in high-risk populations. Early multidisciplinary intervention may improve outcomes in rare oligometastatic disease.

## Introduction

Despite ranking 11th in global cancer incidence, esophageal cancer (EC) carries the 6th highest mortality worldwide, with a disproportionate burden in Asia (1). In China, EC is the 6th most common malignancy with over 90% of Chinese cases classified as esophageal squamous cell carcinoma (ESCC), which is completely different from EC from western countries (2). Known risk factors include tobacco use, alcohol consumption, and region-specific dietary habits — particularly in endemic areas like Chaoshan, where frequent intake of preserved pickles and scalding-hot tea has been linked to increased incidence of ESCC.

The most common metastatic sites of EC are lungs, liver, brains, and bones. Among bone metastases, they frequently occur in the axial skeleton (such as the spine) or proximal ends of the limb bones, while occurrence in the distal ends of the limbs is particularly rare. Indeed, exceptionally rare metastatic sites such as the skin (3), iris (4), cardiac atria (5), and ventricles (6) have also been documented in esophageal cancer cases. To date, only a few cases of EC with metastasis to the fingers have been reported in the literature. However, these typically represent newly developed metastatic lesions detected during post-treatment surveillance. Herein, we uniquely present a case of ESCC initially manifesting as gangrene of the ring finger without prior treatment history.

## Case presentation

**Figure 1:**
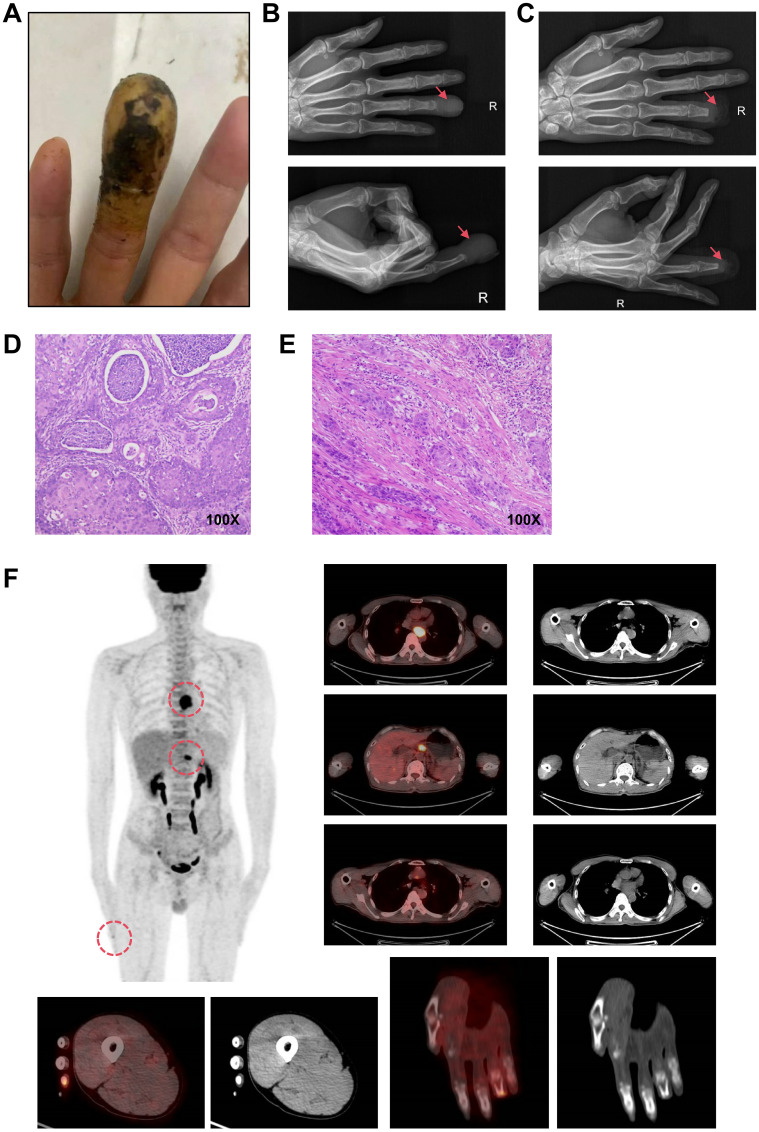
Confirmatory medical examination. **(A)**. Clinical photograph of the right ring finger at initial presentation. **(B)**. Preoperative X-ray of the distal phalanx. **(C)**. Postoperative X-ray following distal phalanx amputation. **(D)**. Histopathological analysis of the amputated distal phalanx. **(E)**. Histopathological analysis of endoscopic biopsies on esophageal lesion. **(F)**. Whole-body 18F-FDG PET-CT images.

**Figure 2:**
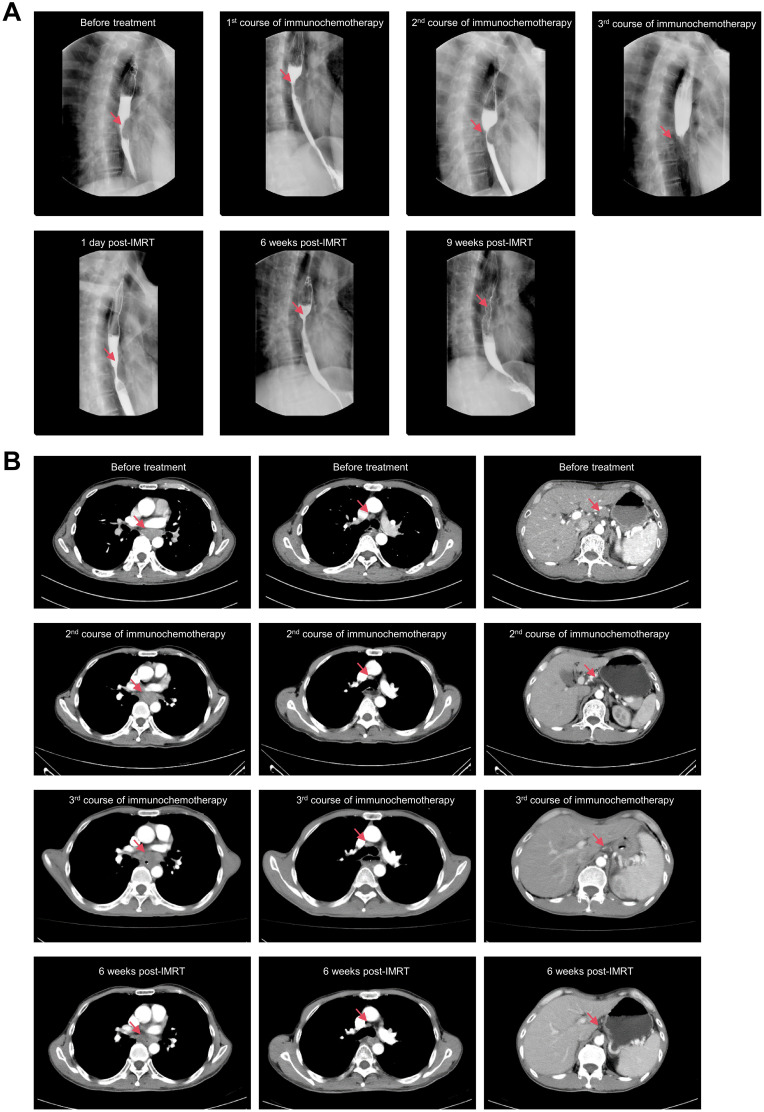
Radiographic evaluations during treatment. **(A)**. X-rays with barium contrast of esophagus. **(B)**. Chest CT scan with contrast.

A 62-year-old Chaoshan male with a lifelong history of preserved pickles and hot tea consumption developed pain in the right ring finger in February 2024, followed by progressive swelling and dark discoloration of the fingertip beginning in April 2024 (**Figure 1A**). Radiographic evaluation at a local hospital in May revealed osteolytic destruction of the distal phalanx in the fourth digit of the right hand with characteristic osseous density abnormalities (**Figure 1B**). On May 23, 2024, distal phalanx amputation of the right ring finger was performed at a local hospital under an initial diagnosis of digital gangrene (**Figure 1C**). However, postoperative histopathological analysis (**Figure 1D**) unexpectedly revealed well-differentiated squamous cell carcinoma with positive surgical margins (carcinoma present at resection margins), supported by characteristic immunohistochemical findings: strong diffuse positivity for pan-cytokeratin (CK, +++), CK5/6 (+++), p63 (+++), and p40 (++); strong membranous expression of epithelial membrane antigen (EMA, +++); focal CD15 reactivity (+); and a markedly elevated Ki-67 proliferative index of 60%. Given that the patient came from a high-risk region for ESCC, where delays in seeking medical attention was common due to cultural normalization of prodromal symptoms (e.g., tolerance of mild dysphagia) coupled with socioeconomic constraints, a chest CT was performed on May 25. CT imaging revealed dilatation of the mid-thoracic esophagus, with marked irregular mural thickening, luminal stenosis with architectural distortion, and mediastinal lymph node metastases. Subsequent endoscopic evaluation identified a circumferential tumor originating at 30–35 cm from the incisors along the left lateral esophageal wall. The lesion exhibited characteristic mucosal abnormalities: coarse surface, friable texture, marked hemorrhagic tendency and luminal narrowing. Histopathological analysis of endoscopic biopsies confirmed moderately differentiated ESCC (**Figure 1E**). Upon rigorous retrospective interrogation of the medical history, the patient recalled that he had experienced intermittent dysphagia and mild thoracodorsal pain one month after the onset of digital pain symptoms, which had been ignored. Further, whole-body 18F-FDG PET-CT (**Figure 1F**) imaging revealed a mid-thoracic esophageal lesion characterized by thickened circumferential wall with luminal stenosis and intense FDG avidity, displaying infiltrative margins encroaching the adjacent descending aorta. Metastatic involvement was also observed in multiple mediastinal lymph node stations, with the largest ones predominantly located in the gastro-hepatic gap. Additionally, postoperative changes at the right ring finger amputation site showed mild residual metabolic activity. The PET-CT also confirmed no other tumor sites beyond the esophageal primary or the distal lesion.Consequently, the Esophageal Cancer Multidisciplinary Team (EC-MDT) confirmed the diagnosis of mid-thoracic esophageal squamous cell carcinoma at stage *T4bN2M1* according to the AJCC(The American Joint Committee on Cancer, AJCC) 8th edition criteria, and recommended initiating immunochemotherapy, followed by response evaluation to determine eligibility for consolidative locoregional therapy (e.g., surgical resection of esophagus and digit or radiochemotherapy). Unfortunately, although the patient’s dysphagia improved after the first cycle of combined immunochemotherapy with tislelizumab, docetaxel, and cisplatin, progressive worsening of symptoms was observed following the second and third treatment cycles while follow-up chest CT also revealed that the lesion was slightly larger than before. Given the stable disease (SD) status and ineligibility for surgical intervention, radiotherapy targeting the primary esophageal lesion and lymph nodes was initiated with planning dose fractionation of 60 Gy in 30 fractions, five days a week. Concurrent weekly cisplatin (30 mg/m²) was administered for 5 cycles during radiotherapy. Following treatment completion, the patient’s dysphagia demonstrated improvement from previous assessments. Follow-up CT revealed a reduction in the size of the esophageal lesion, while mediastinal lymph node metastases remained stable compared to prior imaging.While the surgical intervention effectively addressed the immediate local problem, it crucially did not allow for an assessment of the systemic treatment’s efficacy on the established bony metastasis. The overall therapeutic efficacy was assessed as partial response (PR) per RECIST(Response Evaluation Criteria in Solid Tumors, RECIST) 1.1 criteria (**Figures 2A, B**).

## Discussion

It is estimated that the incidence of finger metastases accounts for less than 0.1% of all bone metastases cases (7). **Table 1** summarizes English literature on digital (finger/toe) metastases identified through a PubMed search. Among the five retrieved cases, four developed metastases 3–24 months post-definitive esophageal cancer treatment (surgery or chemoradiotherapy), with one case occurring during preoperative neoadjuvant chemotherapy. Even though multiple case reports have documented phalangeal metastases in EC during post-treatment surveillance (8–12), to the best of our knowledge, here we represent the first documented case of ESCC with digital gangrene as the first symptom and amputation as the first treatment before the diagnosis of cancer is confirmed. Following a multidisciplinary approach encompassing digital amputation, immunochemotherapy, and localized radiotherapy to the primary lesion, the disease achieved PR according to RECIST 1.1 criteria. Therefore, this case suggests that for patients with oligometastatic ESCC, chemoradiotherapy of the primary lesion combined with surgical resection of the oligometastatic lesions is an effective therapeutic strategy based on systemic systemic treatment.

**Table 1:** Summary of previous studies reporting phalangeal metastases of EC.

Authors	Year	Gender/age	Metastasis location	Metastasis detection timing	Treatment	Primary tumor	PMID
Houston et al. (10)	2000	Male/56	Left ring finger	Occurred 7 months after esophagectomy	phalangectomy	Esophageal basaloid squamous cell carcinoma	10885705
Hsieh et al. (9)	2008	Male/56	Left thumb	Occurred 3 months after esophagectomy	salvage chemo-therapy	Middle thoracic esophageal squamous cell carcinoma	19088530
Atway et al. (11)	2012	Male/38	Right great toe	Occurred 1.5 years after esophagectomy and adjuvant chemotherapy	great toe ampu-tation	Esophageal adenocarcinoma	22153295
Purkayastha et al.	2013	Female/65	Right ring finger	Occurred 2 years after definitive chemoradio-therapy	phalangectomy	Upper thoracic esophageal squamous cell carcinoma	25762887
Chen et al. (12)	2017	Male/44	Right ring finger soft tissue	Occurred during the neoadjuvant chemo-therapy	phalangectomy	Mid-to-lower thoracic esophageal squamous cell carcinoma	28533688

The onset of digital gangrene is commonly associated with identifiable predisposing factors, such as thrombotic events, traumatic injuries, or refractory local infections. In this case, however, the patient developed digital gangrene manifesting as progressive swelling, pain and black discoloration in the absence of discernible triggers. Pathological examination following amputation unexpectedly revealed metastatic squamous cell carcinoma, prompting thorough retrospective clinical evaluation. This interrogation uncovered that the patient developed slight dysphagia and thoracodorsal pain one month after the onset of the finger symptoms, and was ultimately diagnosed with advanced esophageal cancer through a comprehensive diagnostic examination. Consequently, this case also emphasizes that clinicians should broaden the differential diagnostic framework when evaluating unexplained digit lesions, particularly to exclude metastatic EC in high-risk regions. It is very important to maintain heightened clinical vigilance for occult malignancies even in the absence of classic cancer-related symptoms, thereby mitigating risks of delayed diagnosis. However, whether this phenomenon predominantly occurs in ESCC or esophageal adenocarcinoma remains unclear, necessitating further in-depth exploration and mechanistic elucidation.

Actually, the rare association of malignant disease with digital ischemia preceding diagnosis of carcinoma has been reported since 1884 (13). Current evidence suggests that paraneoplastic syndrome, particularly tumor-induced hypercoagulability and autoimmune-mediated vasculopathy, may underlie this association (14–17). Furthermore, this phenomenon is more common in elderly women, and the most common symptom is a gangrenous finger or fingers, as described by Chow et al. (14) and Maurice et al. (15). As a result, when elderly patients present with sudden-onset digital ischemia, particularly when gangrene is present without evidence of embolism, malignancy should be suspected. We recommend active screening for both primary and metastatic cancers to facilitate early disease detection and timely therapeutic intervention.

## Conclusion

This case highlights the potential for digital gangrene to serve as the initial manifestation of ESCC. In high-incidence regions for EC, clinicians must maintain heightened vigilance toward atypical metastatic patterns and optimize therapeutic decision for patients with advanced-stage through multidisciplinary collaboration.

## Data Availability

The original contributions presented in the study are included in the article/supplementary material. Further inquiries can be directed to the corresponding author.
